# Protein-Balanced Dietary Habits Benefit Cognitive Function in Japanese Older Adults

**DOI:** 10.3390/nu15030770

**Published:** 2023-02-02

**Authors:** Keisuke Sakurai, Erika Okada, Saya Anzai, Risako Tamura, Izumi Shiraishi, Noriko Inamura, Satoru Kobayashi, Mikako Sato, Takashi Matsumoto, Kazuyuki Kudo, Yukihiro Sugawara, Tatsuhiro Hisatsune

**Affiliations:** 1 Department of Integrated Biosciences, Graduate School of Frontier Sciences, The University of Tokyo, Kashiwa 277-8562, Japan; 2 Urban Design Center Kashiwanoha (UDCK), Kashiwa 277-0871, Japan; 3 Community Health Promotion Laboratory, Mitsui Fudosan, Co., Ltd., Kashiwa 277-8519, Japan; 4 Research & Development Center, NH Foods Ltd., Tsukuba 300-2646, Japan; 5 Lifestyle Research Office, NH Foods Ltd., Shinagawa, Tokyo 141-6014, Japan

**Keywords:** dietary pattern, daily dietary habits, cluster analysis, discriminant analysis, modeling, mild cognitive impairment, aging

## Abstract

Since daily dietary habits can affect cognitive function, dietary patterns such as the Mediterranean-DASH Intervention for Neurodegenerative Delay diet have been proposed as interventions to slow cognitive decline. However, because dietary habits vary widely among different food cultures, it is necessary to establish dietary pattern intervention methods that are appropriate for each population. Therefore, in this study, the dietary patterns of elderly Japanese individuals were classified using cluster analysis, and their relationship with cognitive function was investigated. We then modeled the dietary patterns and applied them to another cohort of elderly Japanese individuals to determine whether differences in dietary patterns could predict cognitive decline. One hundred and fifty older adults ≥ 65 years of age in the community were recruited. Their daily food intake and cognitive function were measured using the brief-type self-administered diet history questionnaire and Montreal Cognitive Assessment, respectively. K-means cluster analysis identified a high-carbohydrate (HC) dietary pattern with high cereal intake and a protein-balanced (PB) dietary pattern with high intake of legumes, vegetables, seafood, meat, and eggs. Cognitive function was significantly higher in the PB group than in the HC group. Furthermore, to classify the new data into HC and PB patterns, a classification model was created by discriminant analysis using food groups with significantly different intakes among dietary patterns. Next, we recruited 267 new older adults ≥ 65 years of age and measured food intake and cognitive function assessed using the memory performance index score. Individuals with cognitive decline were identified and their detailed cognitive functions were assessed using the neurocognitive index score. Cognitive function was significantly impaired in the HC pattern in both the general elderly and cognitively impaired cohorts. These findings suggest that a dietary pattern of low carbohydrate and high protein intake is associated with good cognitive function in elderly Japanese individuals. Classification by these dietary patterns can predict cognitive reservation in community-dwelling older adults.

## 1. Introduction

Dementia was the seventh leading cause of death worldwide in 2022, with 10 million new cases reported each year [1]. As of 2019, it is estimated that there are approximately 50–60 million people with dementia worldwide, with a predicted increase to approximately 150 million by 2050 [2]. The global societal cost of dementia in 2019 was estimated at USD 1.3 trillion, and the growing number of people with dementia has enormous social and economic consequences [3]. Alzheimer’s disease (AD), the most common cause of dementia, has no treatment to halt or reverse its progression, although some treatments temporarily improve symptoms [4]. AD generally develops after a stage called mild cognitive impairment (MCI), in which there is cognitive impairment beyond the normal cognitive decline associated with aging, but not to an extent that interferes with daily life [5]. According to the Alzheimer’s Disease Neuroimaging Initiative, the transition rate from MCI to AD is estimated to be 16.5% per year [6]. Approximately 24% of people return to normal cognition from MCI. Therefore, it is important to take appropriate measures at the MCI stage at the latest to reduce progression to AD [7].

Currently, no drugs are approved by the United States Food and Drug Administration for the treatment of MCI, and pharmacotherapies targeting the non-dementia stage have not yet been established. Therefore, the focus of treatment is to improve lifestyle risk factors, such as diet and exercise [8]. Diet is considered a modifiable risk factor for the development of dementia. Many nutrients, such as unsaturated fatty acids, polyphenols, tea leaf extracts, caffeine, imidazole dipeptides, and some probiotics, have beneficial effects on the course of cognitive decline in older adults [9,10,11,12,13].

In recent decades, research on the effects of diet on cognitive decline has shifted to dietary patterns that consider nutrient interactions and synergies, rather than the function of individual nutrients [14]. A number of studies examining the relationship between dietary patterns and cognitive function suggest that specific food combinations may have a greater effect than individual nutrients alone [15]. Therefore, establishing a high-quality dietary habit rich in food diversity, rather than a dietary pattern high in a single food such as staple grains, is attracting attention as a dietary strategy for disease prevention [16]. In particular, the Mediterranean diet, Dietary Approaches to Stop Hypertension (DASH) diet, and Mediterranean-DASH Intervention for Neurodegenerative Delay (MIND) diet, which combine these elements to reduce cognitive decline, have been actively studied and associated with lower cognitive decline and risk of AD [17]. These dietary patterns emphasize a balanced intake of a variety of foods, including vegetables, fruits, legumes, fish, and poultry [17].

Although nutritional intervention by changing dietary patterns is a promising approach to suppressing cognitive decline, it is difficult to apply the MIND diet and the Mediterranean Sea diet, which were developed with reference to Western dietary habits, directly to an Eastern country like Japan, because dietary habits vary greatly depending on the dietary culture of each country.

The purpose of this study was to identify dietary patterns that suppress cognitive decline that are easily applicable to elderly East Asian people, like the Japanese, by classifying dietary patterns based on the actual dietary intake of elderly Japanese through cluster analysis and examining differences in cognitive function among dietary patterns. Furthermore, to make the dietary patterns found by cluster analysis classifiable for new data, the classification was modeled by discriminant analysis and applied to a new cohort to confirm the differences in cognitive function among dietary patterns.

## 2. Materials and Methods

### 2.1. Study Design

First, we recruited 150 adults ≥ 65 years of age living in Kashiwa City, a typical suburban city in Japan, to model the dietary pattern classification in the general Japanese elderly population. This population of 150 was named Cohort A. To identify characteristic dietary patterns, cluster analysis was performed using energy intake by food group as a variable, as estimated by the dietary history survey.

Next, to create a valid and reproducible model for classifying new data based on the dietary patterns found by Cohort A, a discriminant analysis model was created based on the estimated energy intake of food groups that differed significantly among the dietary patterns. We recruited 267 new Kashiwa City residents ≥ 65 years of age. This population of 267 was named Cohort B. In Cohort B, a brief cognitive screen test was administered.

In addition, from Cohort B, those with suspected cognitive decline with a cognitive screening score below 60 were selected to undergo additional detailed cognitive tests. This subgroup of Cohort B of 83 individuals who underwent additional cognitive testing was named Cohort B_(CNS)_.

**Figure 1:**
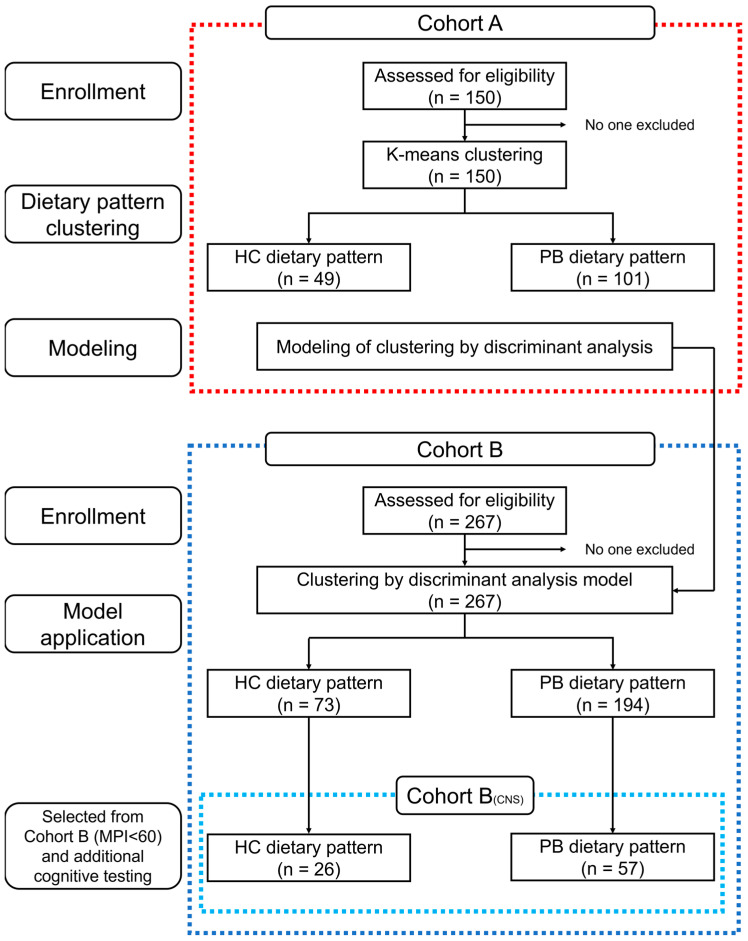
Flow chart showing the number of participants in each phase of Cohorts A, B, and B_(CNS)_.

Finally, a discriminant analysis model was applied to Cohorts B and B_(CNS)_ to classify them by dietary patterns. The flow from the classification of dietary patterns by cluster analysis, modeling of the classification, and application of the model to new data is shown in Figure 1. Informed consent was obtained from all subjects. This study was conducted in accordance with the Declaration of Helsinki and approved by the Ethics Committee of the University of Tokyo (ethical approval code 17-218 and date of approval: January 11 2018).

### 2.2. Participants

#### 2.2.1. Cohort A

We recruited older adults aged over 65 years living in the Kashiwa City area from our volunteer database. To exclude the possibility of reverse causality, whereby dietary patterns are altered by symptoms of dementia, individuals who had already been diagnosed with dementia at the clinic were excluded from the recruitment process. However, a family history of dementia and non-dementia conditions that could affect cognitive function were not considered as exclusion criteria. The 150 individuals comprised Cohort A. Measures in Cohort A included demographic and physical characteristics (age, sex, education, and body mass index (BMI)), cognitive function measured by the Montreal Cognitive Assessment (MoCA), and dietary history in the previous month assessed by the brief-type self-administered diet history questionnaire (BDHQ).

#### 2.2.2. Cohort B

We recruited 267 new participants from our volunteer database. As in Cohort A, these participants were older adults aged over 65 years living in the Kashiwa City area, excluding those with a diagnosis of dementia. The 267 individuals comprised Cohort B. In Cohort B, in addition to a demographic and physical characteristic survey and a dietary history questionnaire with the BDHQ, the Japanese version of the word list memory test was administered as a cognitive screen.

#### 2.2.3. Cohort B_(CNS)_

Cohort B_(CNS)_ is a subgroup of Cohort B who were selected from Cohort B with an MPI score (performance index of the Japanese version of the word list memory test, a cognitive screening test administered in Cohort B) of less than 60 and underwent the Japanese version of the CNS Vital Signs neurocognition test, a more detailed cognitive function test. The reason for the additional testing of this subgroup was to examine in detail the relationship between cognitive function and dietary patterns, especially in those with suspected cognitive decline.

### 2.3. Assessment of Daily Nutritional Intake

Food intake and nutrient intake for all participants were estimated using the BDHQ self-administered dietary history questionnaire. The 80 questions elicit information on the estimated intake of 58 foods and over 100 nutrients [18,19]. The 58 food items were aggregated into 15 food groups (cereals, potatoes, sugars and sweeteners, legumes, green vegetables, other vegetables, fruits, seafood, meat, eggs, milk, fats and oils, confectioneries, beverages, seasonings, and spices). The nutrient intake for each food group was calculated. Food frequency questionnaires (FFQs) are widely used to estimate the long-term food and nutritional intake of the target population. It is recommended that FFQs adapted to the eating habits of the study population be used to avoid cultural and social discrepancies [20]. The BDHQ is an FFQ, optimized for Japanese dietary culture and validated by comparison with semi-weighted dietary records [19].

### 2.4. Evaluations of Cognitive Functions

Cognitive function in Cohort A was assessed using the Japanese version of the MoCA. The MoCA is widely used as a cognitive screening test with high sensitivity and specificity for detecting MCI [21]. In Cohort B, cognitive function was measured using the Japanese version of the word list memory test, rather than the MoCA, to determine whether differences in cognitive function between dietary patterns could be observed using another cognitive test. This cognitive screening test uses an algorithm that considers age, years of education, and race to calculate a score called the Memory Performance Index (MPI) on a scale from 0 to 100 [22,23]. Although it is a simple screening test that assesses the pattern of recall and non-recall of 10 words, it can distinguish between normal cognition and MCI with 96–97% accuracy [23]. In Cohort B_(CNS)_, in addition to the MPI score, cognitive function was assessed in more detail using the Neurocognitive Index (NCI), an overall cognitive measure of the Japanese version of the CNS Vital Signs [24].

### 2.5. Statistical Analyses

The classification of dietary patterns in Cohort A was performed by cluster analysis using the K-means method. Cluster analysis was performed with the variable of estimated energy intake per 1000 kilocalories for each of the 15 food groups, calculated using the BDHQ. The analyst must specify the number of clusters in the K-means method. Based on the elbow method, one of the methods for estimating the appropriate number of clusters [25], two clusters were adopted, where the decreasing rate of the sum of the distances between the cluster center of gravity and each point was sharply smaller in the analysis of this study. Based on the results of the cluster analysis, the classification of dietary patterns was modeled by nonlinear discriminant analysis using Mahalanobis distance with intake of cereals, legumes, green vegetables, other vegetables, seafood, meat, and eggs. These are food groups whose intake differs significantly among the clusters. Participant characteristics and food and nutrient intake were compared across dietary patterns using chi-square tests for categorical variables and Mann–Whitney U tests for continuous variables. Because participant characteristics such as age, sex, years of education, and BMI may influence cognitive function [26,27], an analysis of covariance was performed using these as covariates to compare cognitive function across dietary patterns adjusted for the effects of potential confounders. Data analysis and statistical tests were performed using Python libraries and Bell Curve for Excel ver. 4.01 (Social Survey Research Information Co., Ltd., Tokyo, Japan). Statistical significance was set at *p* < 0.05.

## 3. Results

### 3.1. Identification of the Dietary Patterns by Cluster Analysis

Cluster analysis based on food group intake in Cohort A identified two dietary patterns (Table 1). Forty-nine participants were classified into clusters with a significantly higher cereal consumption (Table 2). This cluster was designated the high-carbohydrate (HC) dietary pattern with high cereal intake. One hundred and one participants were classified into a cluster with significantly higher consumption of legumes, green vegetables, other vegetables, seafood, meat, and eggs (Table 2). This cluster was designated the protein-balanced (PB) dietary pattern with high legume, seafood, meat, and egg intake. Although there were no significant differences in age, years of education, or BMI between the dietary patterns, the HC pattern had a significantly larger proportion of men than the PB pattern (Table 1). Cognitive function evaluated using the MoCA score was significantly lower in the HC group than in the PB group (Table 1). A comparison of nutrient intake by dietary pattern showed no significant differences in energy intake, but the HC pattern had significantly higher carbohydrate intake, whereas the PB pattern had significantly higher protein, fat, ash, and fiber intake (Table 3).

### 3.2. Modeling of Dietary Pattern Classification by Discriminant Analysis

The following discriminant analysis model was created using the intake of food groups, with significant differences among dietary patterns in the cluster analysis of Cohort A as variables. The variables m_1_, m_2_, m_3_, m_4_, m_5_, m_6_, and m_7_ represented the average energy intake per 1000 kcal of cereals, legumes, green vegetables, other vegetables, seafood, meat, and eggs, respectively, in the HC pattern (m_1_ = 402.9, m_2_ = 38.7, m_3_ = 19.1, m_4_ = 23.4, m_5_ = 77.3, m_6_ = 74.0, and m_7_ = 33.9). The PB patterns were likewise n_1_, n_2_, n_3_, n_4_, n_5_, n_6_, and n_7_ (n_1_ = 224.3, n_2_ = 53.2, n_3_ = 26.1, n_4_ = 32.0, n_5_ = 132.0, n_6_ = 100.6, and n_7_ = 45.7). The individuals to be discriminated were x_1_, x_2_, x_3_, x_4_, x_5_, x_6_, and x_7_. The unbiased variance–covariance matrix of energy intake per 1000 kcal of cereals, legumes, green vegetables, other vegetables, seafood, meat, and eggs for the HC group was denoted as S_A_ and the PB group as S_B_.

$$SA_{\;}=\left(\begin{array}{ccccccc} 7232.6 & -597.2 & -213.6 & -296.3 & -646.0 & -646.4 & -192.2\\ -597.2 & 661.1 & 47.0 & 23.0 & 32.0 & -46.6 & 56.4\\ -213.6 & 47.0 & 72.9 & 48.3 & -43.7 & 94.5 & 34.8\\ -296.3 & 23.0 & 48.3 & 88.4 & -25.2 & 111.1 & 12.8\\ -646.0 & 32.0 & -43.7 & -25.2 & 940.6 & 22.4 & -136.1\\ -646.4 & -46.6 & 94.5 & 111.1 & 22.4 & 735.6 & -15.7\\ -192.2 & 56.4 & 34.8 & 12.8 & -136.1 & -15.7 & 405.2 \end{array}\right)$$

$$SB_{\;}=\left(\begin{array}{ccccccc} 3191.6 & -395.6 & -299.0 & -287.1 & -478.3 & -553.7 & -33.9\\ -395.6 & 693.7 & 59.0 & 112.1 & -13.2 & -31.3 & 107.1\\ -299.0 & 59.0 & 142.2 & 74.9 & 72.3 & 63.8 & 19.2\\ -287.1 & 112.1 & 74.9 & 160.6 & 126.2 & 171.6 & 15.9\\ -478.3 & -13.2 & 72.3 & 126.2 & 3299.6 & -30.3 & -121.8\\ -553.7 & -31.3 & 63.8 & 171.6 & -30.3 & 2110.4 & -3.0\\ -33.9 & 107.1 & 19.2 & 15.9 & -121.8 & -3.0 & 488.6 \end{array}\right)$$

(x_1_ − m_1_ x_2_ − m_2_ x_3_ − m_3_ x_4_ − m_4_ x_5_ − m_5_ x_6_ − m_6_ x_7_ − m_7_) S_A_^−1 t^(x_1_ − m_1_ x_2_ − m_2_ x_3_ − m_3_ x_4_ − m_4_ x_5_ − m_5_ x_6_ − m_6_ x_7_ − m_7_) − (x_1_ − n_1_ x_2_ − n_2_ x_3_ − n_3_ x_4_ − n_4_ x_5_ − n_5_ x_6_ − n_6_ x_7_ − n_7_) S_B_^−1 t^(x_1_ − n_1_ x_2_ − n_2_ x_3_ − n_3_ x_4_ − n_4_ x_5_ − n_5_ x_6_ − n_6_ x_7_ − n_7_) (1)

**Figure 2:**
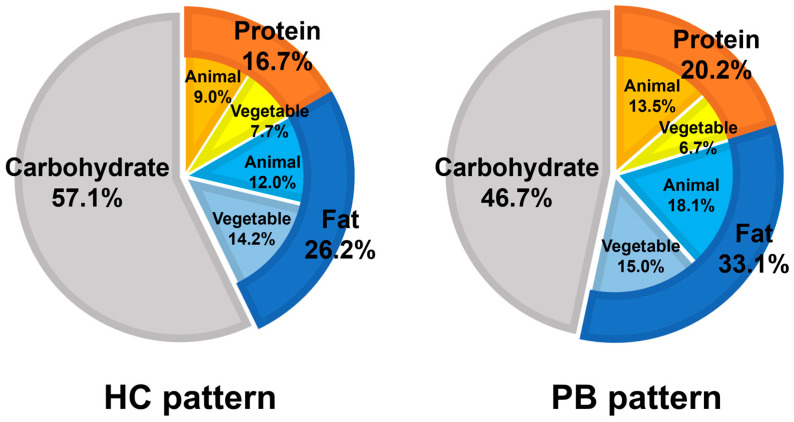
Percentage of energy-based intake of the three macronutrients in the two dietary patterns classified by discriminant analysis model. Data are presented as median values.

If formula (1) < 0 and > 0, the HC and PB patterns, respectively, are determined. The agreement rate between the classification by cluster analysis and discriminant analysis model was 94.0%. In the classification by the discriminant analysis model, as in the classification by cluster analysis, cognitive function was significantly lower in the HC pattern than in the PB pattern (Table 4). In the discriminant analysis model, participants were classified according to the intake of cereals, legumes, green vegetables, other vegetables, seafood, meat, and eggs. However, in addition to these, there were significant differences among dietary patterns in potatoes and dairy products (Table 5). In terms of the percentage of energy-based intake of the three macronutrients, the HC pattern included 57.1% carbohydrates, 16.7% protein (9.0% animal protein; 7.7% vegetable protein), and 26.2% fat (12.0% animal fat; 14.2% vegetable fat). The PB pattern included 46.7% carbohydrates, 20.2% protein (13.5% animal protein; 6.7% vegetable protein), and 33.1% fat (18.1% animal fat; 15.0% vegetable fat) (Figure 2).

### 3.3. Predicting Cognitive Decline by Applying the Dietary Pattern Classification Model

After applying the discriminant analysis model to Cohort B, 73 and 194 participants were classified into the HC and PB patterns, respectively. Although there were no significant differences in sex ratio, years of education, or BMI between dietary patterns, age was significantly higher in the HC group than in the PB group (Table 6). Cognitive function, which was evaluated using the MPI score, was significantly lower in the HC pattern than in the PB pattern, both uncorrected and corrected for covariates (Table 6).

After applying the discriminant analysis model to Cohort B_(CNS)_, which was a selection of individuals from Cohort B with MPI scores ≤ 60, 26 participants were classified in the HC pattern and 57 in the PB pattern. Although there were no significant differences in sex ratio, years of education, or BMI between dietary patterns, age was significantly higher in the HC group than in the PB group (Table 7). Cognitive function, which was evaluated using the NCI score, was significantly lower in the HC pattern than in the PB pattern, both uncorrected and corrected for covariates (Table 7).

## 4. Discussion

Nutritional adjustment by changing daily dietary patterns is attracting attention as a promising approach to delay the progression to AD. However, it is difficult to directly apply dietary patterns that are expected to protect cognitive functions, such as the Mediterranean, DASH, and MIND diets, to the Japanese, whose dietary culture is very different from that of Western countries. Therefore, in this study, we conducted a cluster analysis based on the food group intake of elderly Japanese to compare cognitive functions among dietary patterns with the aim of finding dietary patterns that are suitable for elderly Japanese and help maintain cognitive functions. Cluster analysis revealed patterns of a high cereal diet and a high legume, vegetable, seafood, meat, and egg diet. The HC pattern was associated with significantly lower cognitive function than the PB pattern. A classification model was then created based on the food groups with significant differences between dietary patterns and applied to the new cohort. The results showed that cognitive function was significantly lower in the HC pattern as well as in the cohort from which the model was created.

Legumes, green vegetables, other vegetables, and fish, which have a high intake in the PB pattern dietary habits, are recommended foods in the MIND diet [28]. The Mediterranean and DASH diets slow cognitive decline [29,30]. The MIND diet was created by combining these two diets for neurodegenerative delay. This diet slows cognitive decline with aging, and has been suggested to reduce the risk of AD [31]. In the BDHQ used in this study, all foods classified as legumes could be categorized as soy products. Several studies have shown that cognitive function improves with a higher intake of soy products [32,33]. Research on the neuroprotective effects of soy foods has primarily focused on soy isoflavones. The findings suggest that soy isoflavones may improve general cognitive function [34,35]. Consumption of vegetables, especially green leafy vegetables, is associated with less cognitive decline [36,37]. Green leafy vegetables are rich in carotenoids, flavonoids, and vitamin E, which are considered to reduce the risk of cognitive decline [38]. Fish are rich in long-chain omega-3 fatty acids. Eating fish at least once a week reportedly reduces the risk of dementia [39]. The results of this study are consistent with the findings of previous studies.

The PB pattern is rich in proteins and has a relatively low carbohydrate content. Carbohydrates have a much greater effect on insulin secretion than proteins or fats, and low carbohydrate diets are effective in reducing weight loss, reducing risk factors for cardiovascular disease, and improving type 2 diabetes [40,41,42,43]. Hyperinsulinemia and diabetes are significant risk factors for AD [44]. In a clinical study on MCI, a 6-week low carbohydrate diet intervention significantly reduced fasting blood glucose, fasting insulin, and body weight, and improved verbal memory function [45]. Hyperinsulinemia promotes central nervous system inflammation and neurodegeneration; very low carbohydrate diets were reported to reduce inflammatory factors associated with neurodegeneration [46]. Based on these findings, Krikorian et al. [45] suggested that, in addition to improving energy metabolism, the reduction in neuroinflammation may contribute to improved neurocognitive function in MCI. As for grains, only whole grains are recommended in the MIND diet, but foods classified as grains in the BDHQ are not intended to be whole grains. Refined grains cause blood glucose levels to rise more rapidly and produce more insulin than whole grains [47]. In the HC pattern, a high intake of refined grains may stimulate insulin secretion and associated cognitive decline.

In addition to low carbohydrate intake, high protein intake may protect cognitive function. An increased risk of MCI or dementia has been reported in individuals with low energy intake from protein [48]. It has also been reported that the higher the long-term protein intake, the lower the probability of developing subjective cognitive decline [49]. In particular, animal proteins, including meat, are an excellent source of protein because of their high protein absorption rate, and a high protein diet with a high proportion of meat has been reported to significantly improve cognitive reaction time in randomized intervention trials [50]. In particular, imidazole dipeptide, a histidine-containing dipeptide found in meats such as pork, poultry, and fish, has attracted attention as an animal peptide with cognitive function protective effects [51]. In fact, our RCT studies in humans have shown that mixed consumption of anserine and carnosine [52,53,54,55], as well as anserine alone [13], is beneficial for cognitive decline in older adults. Although the mechanism by which protein intake affects cognitive function is not conclusively known, dietary protein intake provides a source of amino acids necessary for neurotransmitter synthesis. For example, serotonin is synthesized from the essential amino acid phenylalanine, and lower serotonin levels are associated with worse cognitive function [56]. Protein intake may also have indirect effects on cognitive function. Japan is the most aged country in the world, and the prevalence of frailty is known to be higher among adults over the age of 80 years than in other countries [57]. Frailty is described as a vulnerable state in which homeostasis is poorly resolved as a result of the cumulative impairments associated with aging, and is associated with a variety of health problems, including dementia [57,58]. Many studies have reported an inverse association between protein intake and prevalence of frailty [59]. A diet high in protein may be linked to the prevention of frailty and, in turn, to a reduced risk of cognitive decline.

Besides, the PB pattern has a higher intake of fats, especially animal fats, than the HC pattern. Previous studies have shown a positive correlation between cognitive function scores and fat intake, particularly oleic acid intake, in elderly Japanese [10]. Oleic acid is a fatty acid found in animal fats as well as in olive oil. For example, lard, the fat of pork, and het, the fat of beef, contain nearly 50% oleic acid in total fat [60]. Olive oil is a recommended food in the Mediterranean and MIND diets [28], and about 70% of the fat in olive oil is oleic acid [61]. Since olive oil is not as common in Japanese food culture as it is in the West, it is possible that animal fats are replacing the role of olive oil as a source of oleic acid.

It is possible that not only the single effects of these specific foods and nutrients on cognitive function, but also the synergistic effects of multiple nutrients through well-balanced intake of various foods may contribute to the improvement of cognitive function. Dietary diversity, also known as food variety, is a concept related to nutritional intake that has received much attention in terms of disease prevention in recent years and may have a unique impact on health beyond being an indicator of nutritional sufficiency [16]. Previous studies examining the relationship between cognitive function and dietary diversity in elderly subjects have shown that cognitive decline is suppressed as dietary diversity scores increase [62], and that higher scores for recommended food intake are associated with less cognitive decline [63]. Because the nutritional intake capacity of the elderly is poorer than that of younger adults and is more susceptible to the effects of a monotonous diet, it is considered important to ensure that dietary diversity is not compromised to maintain brain function, especially in the elderly [64]. Although the mechanisms related to the effects of dietary diversity on cognitive function are often unclear, it has been suggested that dietary diversity may help maintain health by increasing the diversity of the gut microbiota [65] and ensuring a balance of various bioactive substances [66]. To summarize our discussion thus far, the results of this study may have resulted from a diverse diet that included a variety of recommended foods, in addition to the single effects of specific foods and nutrients that have been scientifically supported to slow cognitive decline through research on diets as treatments.

Nevertheless, our study has some limitations. First, the total number of participants in this observational study was 417, which is a small sample size. However, reliability is ensured by the fact that dietary pattern classifications found in one population were applied to another population, and the same dietary patterns were confirmed to be associated with cognitive decline. Second, this was a cross-sectional study and did not show a causal relationship between dietary patterns and cognitive decline. Therefore, although we excluded people diagnosed with dementia from the participant recruitment, we cannot completely dismiss the possibility of reverse causation, that is, that dietary patterns are altered by symptoms of dementia. In addition, although the same results were obtained after adjusting for covariates in the dietary pattern classification applying the discriminant analysis model, it cannot be ruled out that other unknown confounding factors may be influencing changes in dietary patterns and cognitive function. Third, it remains unclear which nutrients in the two dietary patterns affect cognitive function and their mechanism of action. In particular, foods that differ from the recommended and discouraged foods in the well-studied dietary patterns, such as the Mediterranean, DASH, and MIND diets, should be examined in detail to determine whether they are unique to the elderly Japanese population. Finally, detailed cognitive assessment during the application phase of the classification model was performed only for participants with suspected cognitive decline. Needless to say, it would be more desirable to perform additional, detailed cognitive testing on all subjects. However, since the cognitive function of all subjects in Cohort B was already measured by MPI scores, we considered that limited resources should be spent on subjects who were already exhibiting symptoms of cognitive decline.

Despite these limitations, we believe that this study has made a significant contribution to the goal of establishing a diet that reduces cognitive decline suitable for the elderly Japanese by demonstrating dietary patterns associated with cognitive function in the elderly Japanese. Another important implication of our study is that we created a model that classifies dietary patterns according to the intake of seven food groups, and observed differences in cognitive function between dietary patterns, suggesting that simply examining the intake of certain foods may be used to predict cognitive decline in older adults.

We are currently planning an intervention study to shift older adults with HC patterns to PB patterns to prove the causal relationship between dietary patterns and cognitive function found in this study. Validation with larger sample sizes and additional research on the role of individual nutrients is needed to establish a standard diet therapy for dementia in elderly Japanese individuals.

## 5. Conclusions

Cluster analysis based on the daily dietary intake of Japanese older adults identified the HC dietary pattern with high cereal intake and the PB dietary pattern with high intake of legumes, vegetables, seafood, meat, and eggs, with cognitive function scores significantly higher in the PB dietary pattern. Furthermore, when the dietary pattern classification model based on the results of the cluster analysis was applied to another cohort, cognitive function was significantly higher in the PB dietary pattern as well. These findings suggest that a balanced dietary pattern rich in protein, including legumes, meat, seafood, eggs, and vegetables, is beneficial for good cognitive function in Japanese older adults. Because the evidence from this study was derived from the actual dietary history of Japanese older adults, it may have made an important contribution to the ultimate goal of establishing an easily applicable dietary regimen for Japanese older adults that delays cognitive decline. Another important epidemiological implication is the creation of a dietary pattern classification model that predicts cognitive decline, allowing individuals with suspected cognitive decline to be identified simply by examining their dietary history. For future clinical application, further intervention studies should be conducted to examine cognitive function changes due to shifting dietary patterns.

## Figures and Tables

**Table 1:** Characteristics of Cohort A classified by cluster analysis.

	HC (*n* = 49)	PB (*n* = 101)	*p*-Value
Age	74.0 (70.0–78.0)	72.0 (69.0–76.0)	0.119
Sex (male/female)	24/25	24/77	0.002
Years of education	14.0 (12.0–16.0)	14.0 (12.0–16.0)	0.904
BMI	22.8 (20.8–24.0)	21.9 (20.5–23.8)	0.347
MoCA	26.0 (24.0–27.0)	27.0 (25.0–28.0)	0.031

Data, except for sex, are presented as medians and interquartile ranges. Sex data are presented as the number of participants. Abbreviations are: BMI, body mass index; HC, high-carbohydrate dietary pattern with high cereals intake; and PB, protein-balanced dietary pattern with high legumes, vegetable, seafood, meat, and eggs intake.

**Table 2:** Food group intakes in the two dietary patterns of the Cohort A classified by cluster analysis.

	HC (*n* = 49)	PB (*n* = 101)	*p*-Value
Cereals	383.8 (339.9–429.3)	226.2 (182.4–271.2)	<0.001
Potatoes	15.4 (10.1–28.6)	22.4 (11.3–33.1)	0.119
Sugar, sweeteners	9.5 (5.8–13.0)	10.5 (6.9–14.4)	0.227
Legumes	34.7 (21.1–59.1)	47.0 (39.7–67.6)	<0.001
Green vegetables	17.9 (11.2–25.0)	24.1 (17.5–33.8)	<0.001
Other vegetables	21.2 (18.0–27.9)	30.1 (21.8–40.4)	<0.001
Fruits	47.5 (27.9–62.6)	49.0 (36.7–69.3)	0.160
Seafood	75.1 (55.4–92.7)	124.1 (90.3–170.4)	<0.001
Meat	71.4 (55.7–90.7)	90.0 (69.5–124.1)	<0.001
Eggs	27.5 (21.4–48.1)	47.7 (30.9–56.8)	0.001
Milk	60.8 (25.9–75.0)	63.8 (45.0–88.4)	0.089
Fats, oils	43.7 (31.8–53.1)	50.1 (38.0–62.6)	0.120
Sweets, pastries	71.9 (41.2–106.8)	92.2 (46.6–136.0)	0.105
Refreshing beverages	17.4 (10.0–52.4)	17.2 (8.5–55.2)	0.843
Seasonings, spices	28.8 (15.2–45.0)	27.3 (15.8–40.2)	0.389

Data are presented as median and quartile ranges of energy intake per 1000 kilocalories for each food group. Abbreviations are: BMI, body mass index; HC, high-carbohydrate dietary pattern with high cereals intake; and PB, protein-balanced dietary pattern with high legumes, vegetables, seafood, meat, and eggs intake.

**Table 3:** Nutritional intakes in the two dietary patterns of the Cohort A classified by cluster analysis.

	HC (*n* = 49)	PB (*n* = 101)	*p*-Value
Energy	1947.7 (1563.4–2336.6)	2135.5 (1663.9–2504.5)	0.208
Carbohydrate	134.8 (126.4–150.2)	113.2 (100.9–122.1)	<0.001
Protein	39.9 (37.4–44.6)	50.7 (44.8–55.1)	<0.001
Animal protein	21.4 (19.5–26.5)	33.2 (28.0–38.7)	<0.001
Vegetable protein	17.9 (15.8–19.0)	16.5 (14.9–18.3)	0.031
Fat	27.5 (23.6–31.9)	35.2 (32.5–38.7)	<0.001
Animal fat	13.4 (11.4–15.5)	19.8 (16.7–22.1)	<0.001
Vegetable fat	14.8 (12.1–17.0)	16.4 (13.6–18.7)	0.026
Ash	10.9 (10.4–11.9)	12.6 (11.5–13.9)	<0.001
Total dietary fiber	7.6 (6.4–9.1)	8.9 (7.9–10.4)	<0.001
Soluble dietary fiber	2.0 (1.7–2.4)	2.3 (2.0–2.7)	<0.001
Insoluble dietary fiber	5.4 (4.7–6.3)	6.2 (5.5–7.3)	0.001

Data, except for energy, are presented as median and interquartile range of weight (g) consumed for each food group per 1000 kilocalories. Energy data are presented as the median and quartile range of total energy intake. Abbreviations are: BMI, body mass index; HC, high-carbohydrate dietary pattern with high cereals intake; and PB, protein-balanced dietary pattern with high legumes, vegetables, seafood, meat, and eggs intake.

**Table 4:** Characteristics of Cohort A classified by discriminant analysis model.

	HC (*n* = 40)	PB (*n* = 110)	*p*-Value
Age	75.0 (71.0–78.0)	72.0 (69.0–76.0)	0.008
Sex (Male/Female)	21/19	27/83	0.002
Years of Education	14.0 (12.0–16.0)	14.0 (12.0–16.0)	0.675
BMI	22.4 (20.3–23.3)	22.0 (20.8–24.0)	0.713
MoCA	26.0 (24.0–26.3)	27.0 (24.3–28.0)	0.027

Data, except for sex, are presented as medians and interquartile ranges. Sex data are presented as the number of participants. Abbreviations are: BMI, body mass index; HC, high-carbohydrate dietary pattern with high cereal intake; and PB, protein-balanced dietary pattern with high legumes, vegetables, seafood, meat, and eggs intake.

**Table 5:** Food group intake in the two dietary patterns of the Cohort A classified by discriminant analysis model.

	HC (*n* = 40)	PB (*n* = 110)	*p*-Value
Cereals	399.0 (365.9–459.9)	234.1 (185.8–281.8)	<0.001
Potatoes	13.5 (9.3–26.1)	22.8 (12.1–35.4)	0.025
Sugar, sweeteners	9.9 (6.4–12.9)	10.4 (6.9–14.2)	0.429
Legumes	32.1 (22.0–53.2)	46.1 (38.1–66.7)	0.002
Green vegetables	17.8 (11.2–25.3)	24.0 (17.5–33.6)	<0.001
Other vegetables	20.2 (17.0–27.0)	29.2 (21.2–40.4)	<0.001
Fruits	46.7 (27.7–62.5)	49.8 (36.2–67.4)	0.189
Seafoods	71.0 (55.1–89.4)	119.5 (89.1–164.4)	<0.001
Meat	71.0 (52.4–86.9)	89.6 (69.4–121.3)	<0.001
Eggs	25.9 (17.6–44.9)	47.3 (30.9–56.4)	<0.001
Milk	52.7 (24.6–71.4)	64.7 (45.1–88.8)	0.025
Fats, oils	43.4 (31.0–52.1)	49.8 (38.1–64.3)	0.063
Sweets, pastries	73.0 (42.7–106.8)	78.4 (44.5–129.9)	0.225
Refreshing beverages	15.9 (10.7–52.8)	17.3 (8.6–54.3)	0.737
Seasonings, spices	28.6 (17.8–45.0)	27.5 (15.7–40.5)	0.573

Data are presented as median and quartile ranges of energy intake per 1000 kilocalories for each food group. Abbreviations are: BMI, body mass index; HC, high-carbohydrate dietary pattern with high cereals intake; and PB, protein-balanced dietary pattern with high legumes, vegetables, seafood, meat, and eggs intake.

**Table 6:** Cohort B characteristics classified by the discriminant analysis model.

	HC (*n* = 73)	PB (*n* = 194)	*p*-Value
Age	74.0 (72.0–79.0)	73.0 (71.0–77.0)	0.153
Sex (Male/Female)	31/42	55/139	0.028
Years of Education	14.0 (12.0–16.0)	14.0 (12.0–16.0)	0.724
BMI	21.7 (19.9–23.5)	21.9 (20.8–23.6)	0.285
MPI	57.5 (46.2–62.4)	59.2 (52.0–65.6)	0.026
MPI (corrected)			0.029

Data, except for sex, are presented as medians and interquartile ranges. Sex data are presented as the number of participants. Abbreviations are: BMI, body mass index; HC, high-carbohydrate dietary pattern with high cereals intake; and PB, protein-balanced dietary pattern with high legumes, vegetables, seafood, meat, and eggs intake.

**Table 7:** Cohort B_(CNS)_ characteristics classified by the discriminant analysis model.

	HC (*n* = 26)	PB (*n* = 57)	*p*-Value
Age	79.0 (74.8–81.3)	76.0 (73.0–80.0)	0.035
Sex (Male/Female)	12/14	26/31	0.964
Years of Education	13.0 (12.0–16.0)	14.0 (12.0–16.0)	0.167
BMI	21.7 (19.9–23.8)	21.7 (20.7–23.9)	0.780
MPI	45.3 (40.2–51.6)	52.0 (45.4–56.2)	0.007
NCI	95.0 (87.3–103.0)	100.0 (94.0–105.0)	0.015
MPI (corrected)			0.022
NCI (corrected)			0.042

Data, except for sex, are presented as medians and interquartile ranges. Sex data are presented as the number of participants. Abbreviations are: BMI, body mass index; HC, high-carbohydrate dietary pattern with high cereals intake; and PB, protein-balanced dietary pattern with high legumes, vegetables, seafood, meat, and eggs intake.

## Data Availability

Data supporting the results of this study are available from the corresponding author upon reasonable request.
